# Coexisting forms of malnutrition among under-5 children in Bangladesh: results from 2012 to 13 and 2019 Multiple Indicator Cluster Surveys

**DOI:** 10.1017/S1368980025000448

**Published:** 2025-04-07

**Authors:** Md Ridwan Islam, Md Fuad Al Fidah, Md Mushfiqur Rahman, Tahmeed Ahmed, Sharika Nuzhat

**Affiliations:** 1 Nutrition Research Division, International Centre for Diarrhoeal Disease Research, Bangladesh (icddr,b), Mohakhali, Dhaka, Bangladesh; 2 Clinical and Diagnostic Services, International Centre for Diarrhoeal Disease Research, Bangladesh (icddr,b), Mohakhali, Dhaka, Bangladesh; 3 Office of the Executive Director, International Centre for Diarrhoeal Disease Research, Bangladesh (icddr,b), Mohakhali, Dhaka, Bangladesh

**Keywords:** Pediatric malnutrition, MICS, Under-5 children, Multinomial logistic regression

## Abstract

**Objective:**

Underweight, wasting and stunting are crucial malnutrition indicators responsible for morbidities among children. Data regarding coexisting forms of malnutrition (CFM) are scarce. We aimed to investigate the prevalence and associated factors of CFM across two survey years among under-5 Bangladeshi children.

**Design:**

Cross-sectional study.

**Setting:**

Data were acquired from two rounds of Multiple Indicator Cluster Survey (MICS), Bangladesh conducted in 2012–13 and 2019. *Subjects:* The analysis included 43 946 (2012–13: 20 885; 2019: 23 061) under-5 children.

**Results:**

Binomial proportion test, slope index of inequality and multinomial logistic regression models were used for analysis. The prevalence of CFM was 27·45 % and 18·56 % in 2012–13 and 2019, respectively. A significant decrease in the prevalence of CFM was seen across the surveys (*P*-value < 0·001). Children from urban residence ((2012–13:aOR = 0·70, 95 % CI: 0·64, 0·77); (2019:aOR = 0·71, 95 % CI: 0·65, 0·78), higher maternal education ((2012–13:aOR = 0·28, 95 % CI: 0·24, 0·32); (2019:aOR = 0·28, 95 % CI: 0·24, 0·32), larger size at birth ((2012–13:aOR = 0·62, 95 % CI: 0·52, 0·73); (2019:aOR: 0·60, 95 % CI: 0·50, 0·73) and richest wealth quintile ((2012–13:aOR = 0·25, 95 % CI: 0·22, 0·28); (2019:aOR: 0·30, 95 % CI: 0·27, 0·34)) had lower odds of suffering from CFM compared with their counterparts. Children from poorer quintiles were more influenced by CFM than richer quintiles (Coef.:–0·175, 95 % CI: –0·192, –0·157, *P*-value < 0·001). Higher percentage of CFM was observed among rich families in 2019 compared with 2012–13 (24·50 % and 20·15 %, respectively; *P*-value < 0·001)

**Conclusion:**

The findings of this study should help the researchers and policymakers to understand CFM more clearly and plan prospective studies to explore CFM outcomes. Targeted interventional approaches are needed among parents of rural communities to control the burden of CFM.

Malnutrition is a prevailing and alarming condition that affects millions of under-5 children all over the world, mostly in low-and middle-income countries (LMIC)^(1)^. Every one in five children is at risk of suffering from malnutrition in developing countries like Bangladesh^(1)^. Childhood malnutrition, including undernutrition, is attributable to compromised growth, metabolic and immune dysfunction, higher risk of infection, altered development of brain and cognitive function and various abnormalities^(1,2)^. According to the WHO child growth standards, wasting, stunting and underweight are three of the most critical indicators of malnutrition that can affect a child’s health outcomes and general well-being^(3)^. While wasting and underweight condition is considered to be the result of acute malnutrition, stunting represents chronic malnutrition, mainly causing irreversible damage to the child^(4,5)^. A wasted child becomes too thin for one’s height measured by weight-for-height z score, whereas underweight includes children whose weight is low compared with their age^(3)^. Stunting refers to the condition where a child’s height is less in contrast to their age and is measured by height-for-age z score^(3)^.

According to the WHO factsheet, among the five million under-5 children deaths in the world, about 45 % or 2·4 million deaths can be linked to malnutrition, most of which occurred in LMIC^(6)^. The recent ‘Levels and trends in child malnutrition’ report in 2023 by WHO, UNICEF and World Bank states that around 148 million children are still stunted, whereas 45 million under-5 children are suffering from wasting^(7)^. A staggering number of these children are from Southern Asia and mostly are from resource-poor countries^(7)^. The Bangladesh Demographic and Health Survey 2022 reports that the prevalence of stunting is currently 24 %, which declined from 31 % (Bangladesh Demographic and Health Survey 2017–18), but the proportion still contains a large number of children. Relating to this fact, the prevalence of wasting increased to 11 % from 8 %, and the percentage of underweight remained constant to 22 %^(8)^.

The presence of multiple nutritional dysfunctions is referred to as coexisting forms of malnutrition (CFM)^(9)^. Under-5 children with CFM bear a higher risk of mortality compared with standalone forms of malnutrition^(10)^. It also becomes challenging and difficult to control when multiple forms of malnutrition coexist in the body, resulting in increased health risks compared with a single form of malnutrition^(11)^. *McDonald et al.* published an article in 2013 where they showed that more than half of the malnourished children are suffering from CFM worldwide, and even one unit increase in their anthropometric deficits can increase the risk of death among under-5 children^(12)^. Although children who are suffering from a single form of malnutrition possess a two times higher risk of death compared with non-malnourished children, the risk expands tenfold in children suffering from coexisting forms of undernutrition^(12)^. In Bangladesh, children who are suffering from wasting, stunting and underweight conditions combined have nineteen times higher odds of mortality compared with healthy children^(12)^.

It is evident from previous studies conducted on demographic and health surveys that socio-demographic and socio-economic factors are crucial determinants of child malnutrition^(13–16)^. The prevalence of undernutrition can be attributed to multiple factors such as the mother’s age and height, parent’s education, residence, wealth index and other characteristics^(17,18)^. Although an LMIC like Bangladesh is burdened with a major public health concern like childhood malnutrition, results depicting associated factors of CFM among under-5 children are limited. The primary objective of our analysis was to investigate the prevalence across survey years and important factors that are associated with CFM among under-5 children, where CFM was defined as the presence of any two or all three nutritional deficiencies (wasting, stunting and underweight) in under-5 children.

## Methods

The data used in the current study were obtained from two separate rounds of the Multiple Indicator Cluster Survey (MICS) conducted in Bangladesh. The two survey rounds were 2012–13 (Survey 01) and 2019 (Survey 02). MICS is an important tool for gathering standardised, scientifically sound and nationally representative information. These surveys provide valuable insights on socio-economic standings and various health-related indicators. The survey dataset and findings have been made publicly available on UNICEF’s MICS website at (https://mics.unicef.org/surveys). The current study accessed the children and women datasets from each of Bangladesh’s MICS rounds of 2012–13 and 2019. Both of the surveys used a probability-based sampling method and a two-stage sample selection procedure. Census enumeration areas were identified systematically using the probability proportional to size method. This was followed by the selection of twenty households from each enumeration area (55 120 and 64 400 households for 2012–13 and 2019, respectively). More comprehensive information on the MICS methodology can be found elsewhere^(19,20)^.

**Figure 1.**
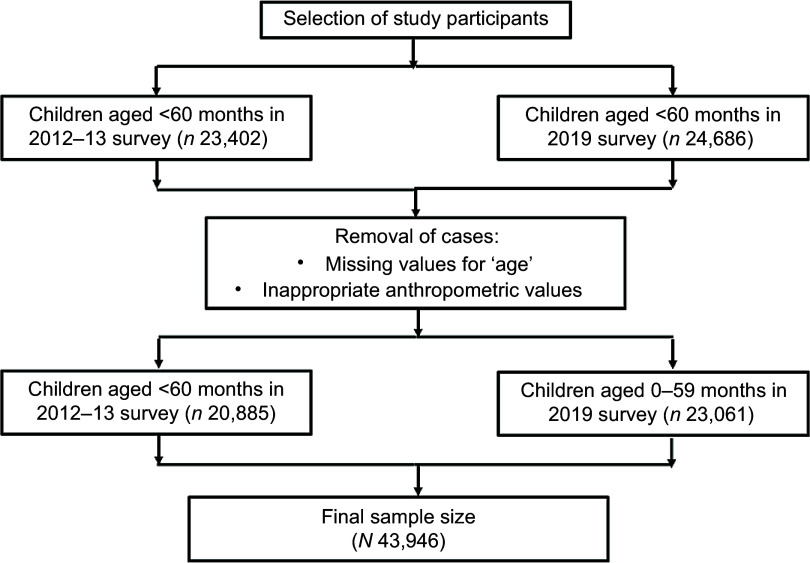
Selection of study participants using combined Bangladesh MICS data from 2012 to 13 and 2019. MICS, Multiple Indicator Cluster Survey.

Children aged < 60 months were included in this study from the publicly accessible datasets and merged with the women ‘wm’ dataset following MICS guidelines for data merging. Cases with ‘missing values’ for age and biologically implausible or inappropriate anthropometric values were excluded (< –5 sd or > 5 sd for WHZ, < –5 sd or > 5 sd for HAZ, < –6 sd or > 5 sd for WAZ)^(21)^. Finally, 20 885 children from 2012 to 13 and 23 061 from 2019 survey rounds were included in the study. The procedure used to select children for this investigation is described in detail in Figure 1.

### Operational definition of the dependent variables

According to the WHO, malnutrition among children can be defined as the excess, deficiency or imbalance in the intake of nutrients and or energy^(1)^. We defined the nutritional indicators as per the WHO child growth standards (2006) in this analysis^(3)^.

*Stunting:* Stunting was defined as height/length-for-age z score < –2 standard deviations (sd)

*Wasting:* Wasting was defined as weight-for-height/length z score < –2 sds

*Underweight:* Underweight was defined as weight-for-age z score < –2 sds

A composite variable ‘Type of malnutrition’ was created by combining stunting, wasting and underweight, which had three categories:

*Absent:* If the participant did not suffer from any of the three defined forms of malnutrition

*Single form of malnutrition (SFM):* If the participant was suffering from any one of the three defined forms of malnutrition

*Coexisting forms of malnutrition (CFM):* If the participant was suffering from more than one of the three defined forms of malnutrition simultaneously

### Independent variables

The current study considered the MICS dataset and selected independent variables on an apriori basis. The variables included are the child’s age (in months), sex (male/female), residence (urban/rural), ever breastfed (yes/no), childhood illness (yes/no), mothers age in years, maternal education, size at birth (average/larger/smaller), maternal media exposure (not exposed/exposed) and wealth index (poorest/poor/middle/rich/richest). For 2012–13 survey, the variable religion (Islam/Hinduism/Buddhism/Christianity/Others) and for 2019 survey, ethnicity (Bengali/Others) and maternal life satisfaction (satisfied/neutral/dissatisfied) were additionally added. A dummy variable (age group in months) was created for the trend test which had five categories: ‘0–12 months’, ‘13–24 months’, ‘25–36 months’, ‘37–48 months’ and ‘49–59 months’ for both survey years.

#### Mother’s education

The variable ‘mother’s education’ was categorised differently in the two survey years. For the survey year 2012–13, the categories were none, primary incomplete, secondary incomplete, secondary complete or higher. However, for the survey year 2019, it was pre-primary or none, primary, secondary, higher secondary or more.

#### Wealth index

The study categorised participants into different socio-economic groups based on a wealth index, which is used to place participants into one of five wealth categories: poorest, poor, middle, rich and richest, by taking inventory of observable household items and amenities^(22,23)^. This method provides an opportunity to gauge relative economic standing without directly measuring income or expenditures^(24,25)^. A dummy variable was also created having two categories, ‘poor-to-middle’ (combining poorest, poor and middle quintiles) and ‘rich’ (combining richest and rich quintiles).

#### Maternal life satisfaction

Maternal life satisfaction was measured using a single item: ‘First, taking all things together, would you say you are very happy, somewhat happy, neither happy nor unhappy, somewhat unhappy or very unhappy?’ Out of five possible responses, ‘very happy’ and ‘somewhat happy’ was categorised as ‘satisfied’, ‘neither happy nor unhappy’ as ‘neutral’ and ‘somewhat unhappy or very unhappy’ as ‘dissatisfied’.

#### Size at birth

Child’s size at birth was measured using a single item: ‘When (name of the child) was born, was (he/she) very large, larger than average, average, smaller than average, or very small?’ The current study categorised ‘very large’ and ‘larger than average’ as ‘large’, ‘average’ as ‘average’ and ‘smaller than average’ and ‘very small’ as ‘small’.

#### Maternal media exposure

‘Maternal media exposure’ was created using the summation of three variables: ‘frequency of reading newspaper or magazine’, ‘frequency of listening to the radio’ and ‘frequency of watching TV’. This variable has two categories as per previous literature: not exposed (0 score) and exposed (> 0 score)^(26)^.

### Statistical analysis

In this study, descriptive statistics were presented as frequency and percentage for categorical variables and median and IQR for continuous variables. Kolmogorov–Smirnov test was conducted on continuous variables, and all were found to be significantly deviated (*P*-value < 0·05) from normal distribution. The χ^2^ goodness-of-fit test was used to assess whether the wealth index of participants with CFM across two survey years was significantly different. The Binomial proportion test was applied to determine the statistical significance of the difference between the prevalence of CFM across two survey years. Kruskal—Wallis test, χ^2^ test of association and Fisher’s exact test were used to evaluate the association between independent and outcome variables. The Cochran–Armitage test for trend was conducted to evaluate changes in the trend of CFM across participant’s age. We used bivariate screening method to select variables for the multinomial logistic regression models. A *P*-value of < 0·05 was considered as significant in the bivariate screening method where only participants with non-missing values were considered. Separate multinomial logistic regression models were developed for each of the survey years (2012–13 and 2019). We adjusted for age and sex to calculate the adjusted OR for each predictor variable, as such missing values for one variable did not have any effect on the adjusted OR for other variables. The adjusted OR (after adjusting for age and sex) and 95 % CI were reported. The slope index of inequality (SII) was used to compare and quantify the association between the wealth index and CFM across both surveys. It quantifies absolute inequality by measuring the difference in an indicator between the most advantaged and most disadvantaged groups while accounting for all intermediate subgroups.^(27)^ To calculate the SII, the entire population is ranked from the most disadvantaged subgroup (rank 0) to the most advantaged subgroup (rank 1), with rankings weighted by each subgroup’s proportional population size. The indicator of interest is then regressed against these midpoint values using an appropriate model. Predicted values of the indicator are calculated for the extremes (rank 0 and rank 1), and the difference between these values gives the SII. An SII value of zero indicates no inequality. For our study, a negative coefficient indicates that CFM is concentrated among the poorest quintile than the richest quintile.^(27)^ A *P*-value of < 0·05 was considered statistically significant (α = 0·05). All descriptive and inferential analysis was done using STATA 17.

## Results

We have included 43 946 children from the two MICS surveys where 20 885 were selected from the year 2012–13 and 23 061 belonged to the year 2019. In 2012–13, 52·35 % (*n* 10 934) children did not have any form of malnutrition, 20·20 % (*n* 4219) children had SFM and 27·45 % (*n* 5732) children had CFM. For 2019, this proportion was 63·57 % (*n* 14 661), 17·86 % (*n* 4119) and 18·57 % (*n* 4281), respectively. Table 1 describes the socio-demographic characteristics among under-5 children and their associations with types of malnutrition across two survey years.

**Table 1.** Association between types of undernutrition and socio-demographic variables across survey years (2012–13, 2019)

Survey year 2012–13									
Characteristics	Total		Types of undernutrition (*n* 20 885)						
			Absent (*n* 10 934)		SFM (*n* 4219)		CFM (*n* 5732)		*P*-value
	Median	IQR	Median	IQR	Median	IQR	Median	IQR	
Age median (IQR)^*^	31	15–45	28	12–45	30	16–44	34	20–46	**< 0·001**
	*n*	%	*n*	%	*n*	%	*n*	%	
Sex (female) *n* (%)	10 161	48·65	5374	49·15	2033	48·19	2754	48·05	0·318
Residence (rural) *n* (%)	17 557	84·07	8979	82·12	3607	85·49	4971	86·72	**< 0·001**
Ever breastfed (yes) *n* (%)	20 463	98·04	10 736	98·23	4133	98·03	5594	97·68	0·054
Childhood illness (yes) *n* (%)^**^	6098	29·2	3201	29·28	1205	28·56	1692	29·52	0·564
Mothers age in years									**< 0·001**
15–19	1230	6·03	643	6·02	255	6·19	332	5·94	
20–24	5932	29·10	3218	30·14	1162	28·19	1552	27·77	
25–29	6622	32·48	3487	32·66	1343	32·58	1792	32·07	
30–34	3599	17·65	1849	17·32	745	18·07	1005	17·98	
35–39	1994	9·78	1022	9·57	399	9·68	573	10·25	
40–44	715	3·51	329	3·08	152	3·69	234	4·19	
45–49	295	1·45	129	1·21	66	1·60	100	1·79	
Maternal education									**< 0·001**
None	5252	25·15	2335	21·36	1113	26·38	1804	31·47	
Primary incomplete	3030	14·51	1379	12·61	661	15·67	990	17·27	
Primary complete	3219	15·41	1531	14·00	713	16·90	975	17·01	
Secondary incomplete	7056	33·79	4044	36·99	1382	32·76	1630	28·44	
Secondary complete or higher	2328	11·15	1645	15·04	350	8·30	333	5·81	
Religion									0·069
Islam	18 491	88·54	9681	88·54	3734	88·50	5076	88·56	
Hinduism	1670	8·00	903	8·26	316	7·49	451	7·87	
Buddhism	492	2·36	237	2·17	113	2·68	142	2·48	
Christianity	111	0·53	61	0·56	28	0·66	22	0·38	
Others	121	0·58	52	0·48	28	0·66	41	0·72	
Size at birth (*n* 9181)									**< 0·001**
Average	5801	63·18	3360	65·59	1151	64·12	1290	57·00	
Larger	1297	14·13	870	16·98	218	12·14	209	9·24	
Smaller	2083	22·69	893	17·43	426	23·73	764	33·76	
Maternal media exposure (*n* 13 995)^***^									**< 0·001**
Not exposed	5875	41·98	2945	37·67	1243	44·83	1687	49·54	
Exposed	8120	58·02	4872	62·33	1530	55·17	1718	50·46	
Wealth index									**< 0·001**
Poorest	6260	29·97	2721	24·89	1360	32·24	2179	38·01	
Poor	4509	21·59	2168	19·83	952	22·56	1389	24·23	
Middle	3771	18·06	2004	18·33	758	17·97	1009	17·60	
Rich	3415	16·35	2010	18·38	651	15·43	754	13·15	
Richest	2930	14·03	2031	18·58	498	11·80	401	7·00	
Survey year 2019									

Characteristics	Total		Types of undernutrition (*n* 23 061)						
			Absent (*n* 14 661)		SFM (*n* 4119)		CFM (*n* 4281)		*P*-value
	Median	IQR	Median	IQR	Median	IQR	Median	IQR	
Age median (IQR)^*^	30	15–44	29	13–45	29	16–43	32	18–45	**< 0·001**
	*n*	%	*n*	%	*n*	%	*n*	%	
Sex (female) *n* (%)	11 133	48·28	7107	48·48	1937	47·03	2089	48·80	0·194
Residence (rural) *n* (%)	18 773	81·41	11 749	80·14	3382	82·11	3642	85·07	**< 0·001**
Ever breastfed (yes) *n* (%) (*n* 13 612)	13 016	95·62	8347	95·90	2407	95·59	2262	94·64	**0·029**
Childhood illness (yes) *n* (%)^**^	8148	35·33	5147	35·11	1405	34·11	1596	37·28	**0·006**
Maternal age (*n* 22 759)									**< 0·001**
15–19	1690	7·43	1088	7·51	314	7·74	288	6·83	
20–24	6811	29·93	4353	30·06	1224	30·16	1234	29·25	
25–29	6637	29·16	4310	29·76	1147	28·27	1180	27·97	
30–34	4668	20·51	2957	20·42	816	20·11	895	21·21	
35–39	2183	9·59	1323	9·14	416	10·25	444	10·52	
40–44	575	2·53	339	2·34	100	2·46	136	3·22	
45–49	195	0·86	112	0·77	41	1·01	42	1·00	
Maternal education									**< 0·001**
Pre-primary or none	2591	11·24	1341	9·15	544	13·21	706	16·49	
Primary	5555	24·09	3172	21·64	1107	26·88	1276	29·81	
Secondary	11 334	49·15	7483	51·04	1932	46·90	1919	44·83	
Higher secondary or more	3581	15·53	2665	18·18	536	13·01	380	8·88	
Ethnicity (Bengali) *n* (%)	22 543	97·75	14 347	97·86	3994	96·67	4202	98·15	**< 0·001**
Caregiver of another child (Yes) *n* (%) (*n* 18 549)	12 535	67·58	7566	66·07	2345	69·13	2624	70·82	**< 0·001**
Wealth index									**< 0·001**
Poorest	5750	24·93	3094	21·10	1213	29·45	1443	33·71	
Poor	4831	20·95	2895	19·75	918	22·29	1018	23·78	
Middle	4345	18·84	2838	19·36	736	17·87	771	18·01	
Rich	4303	18·66	2985	20·36	670	16·27	648	15·14	
Richest	3832	16·62	2849	19·43	582	14·13	401	9·37	
Maternal life satisfaction (*n* 22 752)									**< 0·001**
Satisfied	19 751	86·81	12 787	88·31	3478	85·75	3486	82·67	
Neutral	2291	10·07	1287	8·89	442	10·90	562	13·33	
Dissatisfied	710	3·12	405	2·80	136	3·35	169	4·01	
Size at birth (*n* 10 920)									**< 0·001**
Average	7698	70·49	5098	71·94	1384	70·54	1216	64·96	
Larger	1240	11·36	922	13·01	186	9·48	132	7·05	
Smaller	1982	18·15	1066	15·04	392	19·98	524	27·99	
Maternal media exposure (*n* 22 749)									**< 0·001**
Not exposed	8471	37·24	4924	34·01	1679	41·41	1868	44·29	
Exposed	14 278	62·76	9552	65·99	2376	58·59	2350	55·71	

SFM, single form of malnutrition; CFM, coexisting form of malnutrition. Statistically significant p-values are indicated in bold.

* Kruskal–Wallis test.

** Childhood illness: diarrhoea and cough (for 2012–13); diarrhoea, fever and cough (for 2019).

*** Fisher’s exact test.

### Socio-demographic characteristics

During the survey year 2012–13, the median (IQR) age was higher among the CFM group (34 (20–46) months) compared with the non-malnourished group where the association was statistically significant (28 (12–45); *P*-value < 0·001). Several factors were found to be significantly associated with different types of malnutrition, including residence, maternal age, maternal education, size at birth, maternal media exposure and wealth index. Most of the children (84·07 %) were from rural areas, which was also the case among each type of malnutrition. Additionally, around 60 % of mothers in all groups fell into the age categories of 20–24 or 25–29, with over 33·79 % not completing secondary education. In the CFM group, specifically, 31·47 % of mothers had no formal education, and 50·46 % was exposed to media. Regarding the wealth index, 29·97 % belonged to the poorest group, which was true for each of the malnutrition groups.

In the survey year 2019, several factors were found to be significantly associated with malnutrition types, including the age of child, residence, size at birth, breastfeeding history, childhood illness, maternal age, maternal education, ethnicity, caregiver of other children, maternal life satisfaction, maternal media exposure and wealth index. Among children with CFM, the median (IQR) age was 32 (18–45) months, with 27·99 % being smaller in size at birth. Furthermore, 85·07 % of these children lived in rural areas, and 33·71 % belonged to the poorest wealth index category. Overall, 95·62 % of children were breastfed at some point before the survey, while among CFM cases, it was 94·64 %. More than half of the mothers in each type of malnourished children belonged to the 20–24 years or 25–29 years age group.

### Variations in CFM prevalence across surveys

**Figure 2.**
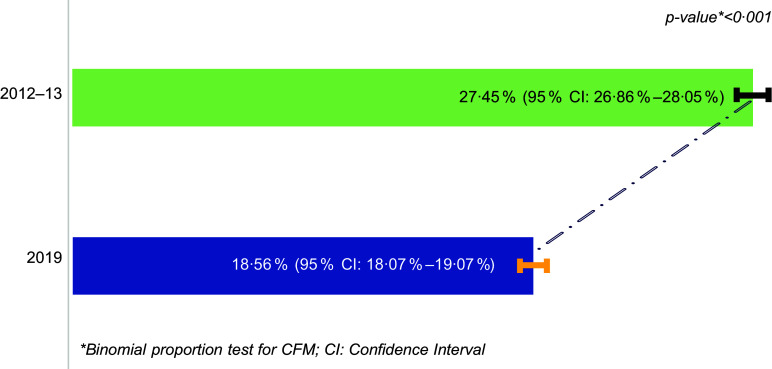
Prevalence of CFM across survey years (2012–13, 2019). CFM, coexisting forms of malnutrition.

Figure 2 shows a decrease in the prevalence of CFM from 27·45 % (95 % CI: 26·86 %, 28·05 %; *n* 5732) in the 2012–13 survey to 18·56 % (95 % CI: 18·07 %, 19·07 %; *n* 4281) in 2019, which was statistically significant (*P*-value < 0·001).

**Figure 3.**
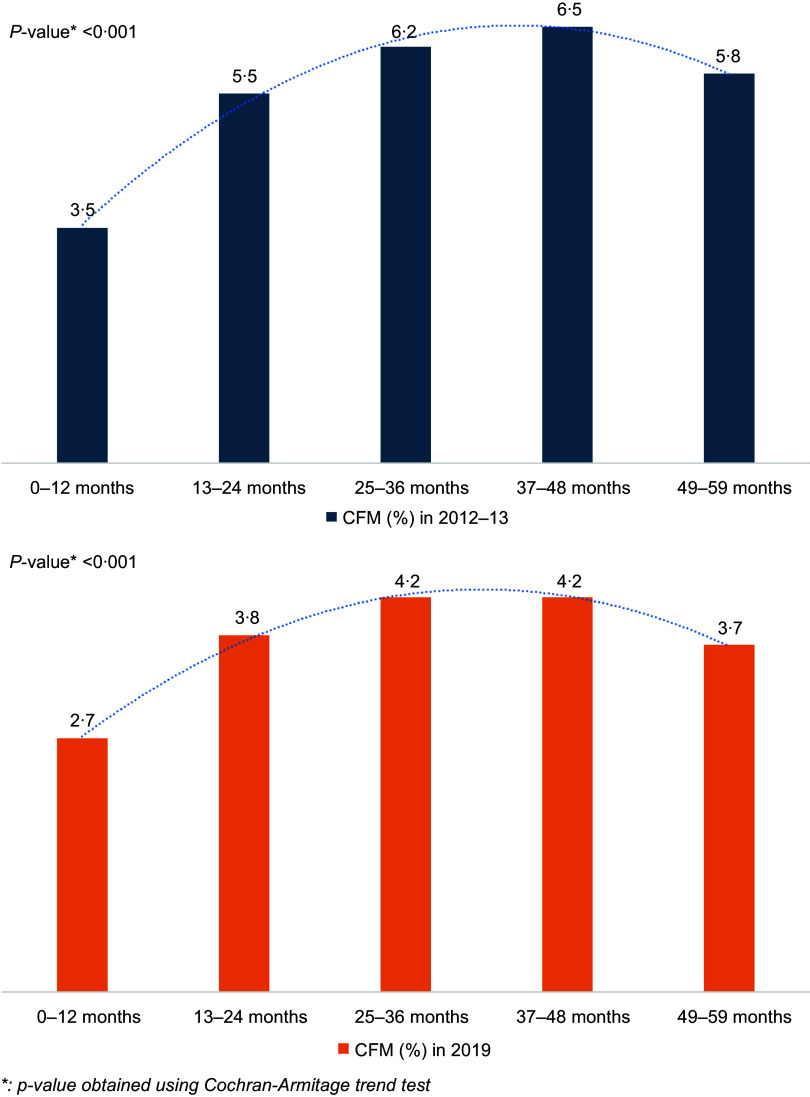
Trend of CFM across participant’s age groups in survey years (2012–13, 2019). CFM, coexisting forms of malnutrition.

Figure 3 shows a statistically significant rise in the trend of CFM from 3·5 % among participants aged 0–12 months to 5·8 % among 49–59 months in the survey year 2012–13. However, the maximum proportion was 6·5 % in the age group 37–48 months followed by 6·2 % in 25–36 months. A similar rising trend was also observed in the survey year 2019 where the proportion of CFM raised from 2·7 % to 3·7 % with a maximum of 4·2 % in age groups 25–36 months and 37–48 months.

**Figure 4.**
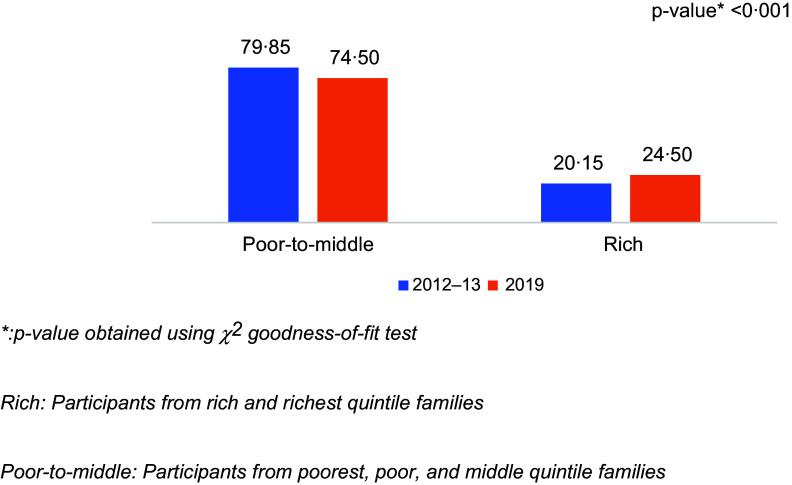
Proportion of wealthier quintile families among participants with CFM. CFM, coexisting forms of malnutrition.

Figure 4 represents the distribution of wealth index among participants who were suffering from CFM across both survey years. Higher percentage of CFM was observed among rich families in 2019 compared with 2012–13 (24·50 % and 20·15 %, respectively) which was significantly different (*P*-value < 0·001).

### Slope index of inequality analysis

**Figure 5.**
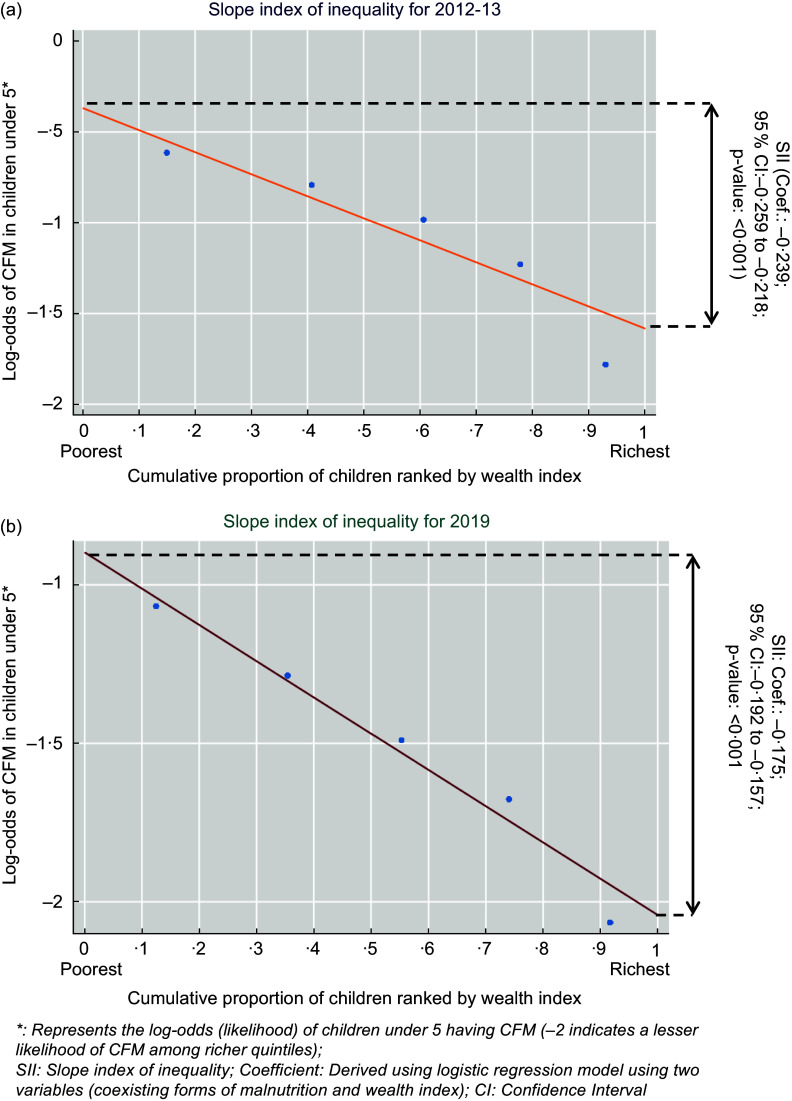
Slope index of inequality (SII) for the prevalence of coexisting forms of malnutrition according to wealth quintiles across survey years; (a) for 2012–13; (b) for 2019. CFM, coexisting forms of malnutrition.

The SII is presented in Figure 5. The negative values of the coefficient for survey years 2012–13 and 2019 (Coef.: –0·239, 95 % CI: –0·259, –0·218; Coef.: –0·175, 95 % CI: –0·192, –0·157 respectively) indicate that belonging to richer quintile families was significantly associated with lesser likelihood of under-5 children suffering from CFM.

### Multinomial logistic regression analysis

Multinomial logistic regression models were constructed after adjusting for the age and sex of the child across both the survey years in Table 2. In the survey year 2012–13, the model showed that place of residence, maternal age in years, maternal education, size at birth, maternal media exposure and wealth index had significant effects on the type of malnutrition. The children living in urban areas had lower odds of CFM (aOR = 0·70, 95 % CI: 0·64, 0·77, *P*-value < 0·001) and SFM (aOR = 0·78, 95 % CI: 0·71, 0·86, *P*-value < 0·001) when compared with children without malnutrition that are living in rural areas. Mothers age group (in years) up to 35–39 were found to have lower odds of CFM, which was statistically significant. Considering the size of the child at birth, if the child was of larger size, the odds for CFM (aOR = 0·62, 95 % CI: 0·52, 0·73, *P*-value < 0·001) and SFM (aOR = 0·73, 95 % CI: 0·62, 0·85, *P*-value < 0·001) decreased, but if it was smaller, the odds of CFM (aOR = 2·32, 95 % CI: 2·06, 2·61, *P*-value < 0·001) and SFM (aOR = 1·42, 95 % CI: 1·24, 1·62, *P*-value < 0·001) increased. Moreover, when mothers completed secondary level education, the odds of CFM decreased (aOR = 0·28, 95 % CI: 0·24, 0·32, *P*-value < 0·001) along with SFM (aOR = 0·45, 95 % CI: 0·40, 0·52, *P*-value < 0·001). Also, when considering maternal media exposure, if the mother was exposed to media, the odds of CFM decreased (aOR = 0·61, 95 % CI: 0·56, 0·66, *P*-value < 0·001) compared with those with no media exposure. The results from Table 2 also showed that when the wealth index increased to be on the richer side, the odds of CFM and SFM decreased compared with those with no malnutrition and the poorest group, which was statistically significant.

**Table 2.** Age- and sex-adjusted multinomial logistic regression models across two survey years (2012–13, 2019) presenting the association between predictor variables and types of malnutrition

Survey year 2012–13							
		SFM			CFM		
Predictor variables		aOR	95 % CI	*P*-value	aOR	95 % CI	*P*-value
Residence	Rural	Ref					
	Urban	0·78	0·71, 0·86	**< 0·001**	0·70	0·64, 0·77	**< 0·001**
Mother’s age in years	15–19	Ref					
	20–24	0·87	0·74, 1·02	0·083	0·79	0·68, 0·92	**0·002**
	25–29	0·91	0·77, 1·07	0·248	0·80	0·69, 0·93	**0·003**
	30–34	0·95	0·80, 1·12	0·519	0·83	0·71, 0·97	**0·019**
	35–39	0·91	0·75, 1·10	0·323	0·84	0·70, 0·99	**0·040**
	40–44	1·07	0·84, 1·36	0·608	1·03	0·83, 1·28	0·795
	45–49	1·17	0·84, 1·64	0·353	1·10	0·82, 1·48	0·544
Maternal education	None	Ref					
	Primary incomplete	1·01	0·90, 1·14	0·845	0·95	0·86, 1·05	0·323
	Primary complete	0·99	0·88, 1·11	0·828	0·86	0·77, 0·95	**0·003**
	Secondary incomplete	0·73	0·66, 0·80	**< 0·001**	0·55	0·50, 0·59	**< 0·001**
	Secondary complete or higher	0·45	0·40, 0·52	**< 0·001**	0·28	0·24, 0·32	**< 0·001**
Size at birth (*n* 9181)	Average	Ref					
	Larger	0·73	0·62, 0·85	**< 0·001**	0·62	0·52, 0·73	**< 0·001**
	Smaller	1·42	1·24, 1·62	**< 0·001**	2·32	2·06, 2·61	**< 0·001**
Maternal media exposure (*n* 13 995)	Not exposed		Ref				
	Exposed	0·74	0·68, 0·81	**< 0·001**	0·61	0·56, 0·66	**< 0·001**
Wealth index	Poorest	Ref					
	Poor	0·88	0·80, 0·97	**0·014**	0·81	0·74, 0·88	**< 0·001**
	Middle	0·76	0·68, 0·85	**< 0·001**	0·64	0·56, 0·79	**< 0·001**
	Rich	0·65	0·58, 0·73	**< 0·001**	0·47	0·43, 0·52	**< 0·001**
	Richest	0·49	0·44, 0·55	**< 0·001**	0·25	0·22, 0·28	**< 0·001**

Survey year 2019							
		SFM			CFM		
Predictor variables		aOR	95 % CI	*P*-value	aOR	95 % CI	*P*-value
Residence	Rural	Ref					
	Urban	0·88	0·80, 0·96	**0·005**	0·71	0·65, 0·78	**< 0·001**
Ever breastfed (*n* 13 612)	No	Ref					
	Yes	1·07	0·86, 1·33	0·556	0·96	0·78, 1·19	0·721
Childhood illness	No	Ref					
	Yes	0·96	0·89, 1·03	0·242	1·14	1·06, 1·23	**< 0·001**
Mother’s age in years (*n* 22 759)	15–19	Ref					
	20–24	0·97	0·84, 1·12	0·696	0·98	0·84, 1·13	0·761
	25–29	0·92	0·80, 1·06	0·252	0·93	0·80, 1·08	0·323
	30–34	0·95	0·82, 1·11	0·529	1·01	0·87, 1·18	0·873
	35–39	1·09	0·92, 1·29	0·347	1·11	0·93, 1·32	0·244
	40–44	1·02	0·79, 1·32	0·899	1·29	1·10, 1·64	**0·041**
	45–49	1·26	0·86, 1·84	0·239	1·19	0·82, 1·75	0·362
Maternal education	Pre-primary or none	Ref					
	Primary	0·86	0·76, 0·97	**0·014**	0·78	0·70, 0·87	**< 0·001**
	Secondary	0·64	0·57, 0·71	**< 0·001**	0·50	0·45, 0·55	**< 0·001**
	Higher secondary or more	0·50	0·43, 0·57	**< 0·001**	0·28	0·24, 0·32	**< 0·001**
Ethnicity	Others	Ref					
	Bengali	0·70	0·57, 0·86	**< 0·001**	1·19	0·93, 1·52	0·177
Caregiver of another child (*n* 18 549)	No	Ref					
	Yes	1·17	1·08, 1·27	**< 0·001**	1·25	1·15, 1·35	**< 0·001**
Size at birth (*n* 10 920)	Average	Ref					
	Larger	0·74	0·63, 0·88	**< 0·001**	0·60	0·50, 0·73	**< 0·001**
	Smaller	1·18	1·21, 1·57	**< 0·001**	2·11	1·87, 2·39	**< 0·001**
Maternal media exposure (*n* 22 749)	Not exposed	Ref					
	Exposed	0·73	0·68, 0·78	**< 0·001**	0·65	0·60, 0·69	**< 0·001**
Wealth index	Poorest	Ref					
	Poor	0·81	0·73, 0·89	**< 0·001**	0·75	0·69, 0·83	**< 0·001**
	Middle	0·66	0·60, 0·74	**< 0·001**	0·58	0·53, 0·65	**< 0·001**
	Rich	0·57	0·51, 0·64	**< 0·001**	0·47	0·42, 0·52	**< 0·001**
	Richest	0·52	0·47, 0·58	**< 0·001**	0·30	0·27, 0·34	**< 0·001**
Maternal life satisfaction (*n* 22 752)	Neutral	Ref					
	Satisfied	0·79	0·71, 0·89	**< 0·001**	0·63	0·57, 0·70	**< 0·001**
	Dissatisfied	0·98	0·78, 1·22	0·853	0·94	0·77, 1·16	0·581

Reference level: Type of malnutrition ‘absent’. CFM, coexisting forms of malnutrition. Statistically significant p-values are indicated in bold.

In Table 2, when considering the survey year 2019, we observed that the findings of predictor variables such as residence, mother’s age, maternal education, child’s size at birth, maternal media exposure and wealth index were similar to the survey year 2012–13. Also, if the caregiver had another child that increased the odds of both malnutrition categories (CFM: aOR = 1·25, 95 % CI: 1·15, 1·35, *P*-value < 0·001; SFM: aOR = 1·17, 95 % CI: 1·08, 1·27, *P*-value < 0·001), while childhood illness increased the odds of CFM (aOR = 1·14, 95 % CI: 1·06, 1·23, *P*-value < 0·001). However, if the mother is satisfied with her life, then it decreases the odds of both (CFM: aOR = 0·63, 95 % CI: 0·57, 0·7, *P*-value < 0·001; SFM: aOR = 0·79, 95 % CI: 0·71, 0·89, *P*-value < 0·001).

## Discussion

This study presents the prevalence of CFM in Bangladesh, its prevalence across both survey years (2012–13, 2019) and related factors affecting CFM. In both the survey years, we could see that the child’s residence, mother’s age, maternal education, size at the time of birth, mother’s exposure to media and wealth index affected CFM similarly. Although CFM presented a decreasing prevalence from years 2012–13 to 2019, the overall prevalence of this nutritional disorder is still high in the country. Further analysis revealed that children from poorer families suffered from CFM more compared with the richer families in both survey years.

Bangladesh, a South Asian country, is burdened with a high prevalence of wasting, underweight and stunting among under-5 children^(8)^. For example, in case of severe wasting, more than 0·3 million under-5 children reside in the country, which is one of the major positions among other Asian countries^(28)^. So, it is no surprise that the prevalence of CFM is also high in the country. In our study, we found that CFM exhibits a downward prevalence across the survey years. This is mostly due to the fact that the Bangladesh government has taken the burden of malnutrition among children seriously. The National Nutrition Service, Institute of Public Health Nutrition (IPHN), non-profit research organisations like icddr,b are working together to achieve the Health, Population and Nutrition Sector Program (HPNSP) target to reduce the prevalence of malnutrition in the country^(8)^. The prevalence of wasting, which was precariously high in 2011 at 16 %, descended to 11 % in 2022^(8)^. Also, the prevalence of stunting and underweight dropped to 24 % and 22 % in 2022 from the year 2011, where the percentages were 41 % and 36 %, respectively^(8)^. The government has also taken multiple steps countrywide and integrated guideline-based management protocols in the hospitals to combat malnutrition in children^(28–30)^. For example, a recent study found that the physicians in government hospitals had adequate knowledge and a positive attitude while treating malnourished children^(28)^. All of these factors might have influenced the decreasing prevalence of CFM in the country.

Community-level socio-demographic and economic factors play a vital role in the prevalence of malnutrition^(31–33)^. Characteristics like socio-economic condition, parents’ educational status, place of residence, childhood illness and several other factors can affect the nutritional status of a child in developing countries^(32–34)^. A study conducted using multiple Bangladesh Demographic and Health Survey surveys (1996–1997, 1999–2000, 2004, 2007 and 2011) examined the trend of childhood malnutrition and the impact maternal education has on it. The authors reported a consistently significant association where lower maternal education favours childhood malnutrition^(35)^. Another study conducted in Bangladesh also supports this finding^(36)^. A 2023 study conducted in Bangladesh found that parental education and occupation had a profound impact on undernutrition among under-5 children in both urban and rural areas of Bangladesh. The findings indicated that children whose fathers worked low-paying jobs, for example, in the agricultural field, were more likely to experience wasting, stunting, or underweight conditions^(36)^. Another Bangladeshi survey also reported that children from the poorest quintiles had three times higher odds of getting affected by wasting^(33)^. A household’s low economic status directly affects its purchasing power, which in turn influences the availability of nutritious food, resulting in food insecurity^(37)^. Households that face social and economic challenges can be associated with decreased physical growth, mostly due to facts like poor food consumption, chronic illnesses, unhygienic lifestyle, insufficient access to pure water, etc.^(31)^. In both our multinomial logistic regression analysis and SII, we have observed that CFM was more likely to be associated with children emerging from poorer families compared with children from richer families. These findings can be justified by the above discussion. Additionally, another study conducted among Bangladeshi under-5 children using nationally representative data reported that CFM is favoured by families that belong to lower wealth index quintiles^(38)^. An interesting finding from our analysis showed that over the years CFM has increased among richer families, though the percentage is still low. A significant rise in CFM was observed in the year 2019 compared with the year 2012–13. A recent paper from Bangladesh showed that stunting was present among children whose mothers were engaged in different income-generating activities^(39)^. It can be hypothesised that as both father and mother are participating in earning activities in rich families the children are left at home to unskilled caregivers who have little knowledge about nutrition. The daycare system is also underdeveloped in an LMIC country like Bangladesh. These factors might be affecting the rise of malnutrition among rich families which needs further evaluation.

When it comes to the topic of childhood malnutrition, the urban–rural divergence has remained persistent over the years^(40)^. This pertains to the difference in socio-economic status, poor accessibility to healthcare facilities, medicine, education and various other factors^(33)^. Multiple studies have reported that rural children are more prone to nutritional deficiency and tend to become malnourished, which corresponds to our findings^(36,40)^. Our study also indicated that maternal age and education are important predictors of CFM, and if the mother was older and received more education, the odds of her children suffering from CFM decreased. A Bangladeshi study found that children from illiterate fathers or mothers were more likely (approximately 20–30 %) to remain underweight or stunted compared with parents who received higher education^(33)^. This was coherent with earlier studies conducted in Bangladesh, which found a significant impact of parenteral education on both SFM and CFM^(35,36,38)^. Mothers who are older and educated possess a clear perception and greater understanding regarding the nutrition and health of the children, healthcare-seeking behaviour, improved care of the child, washing, hygiene and sanitation practices. So, it is understandable that these factors are affecting CFM among children. Also, children born with low birth weight are more likely to suffer from different forms of malnutrition, which is consistent with the findings from our study^(41)^. Previous works of literature found that mothers who are exposed to media have better awareness regarding healthcare practices and can significantly decrease malnutrition through household actions^(26,42)^. This corresponds to our study results as well. Children whose mothers were happy or satisfied with their lives had lower odds of suffering from CFM, according to our study. An article presented that mothers who were unmarried or cohabiting and had low family incomes were more likely to have malnourished children^(43)^. Similarly, poverty-stricken families have lower life satisfaction, which can affect the growth of their children. Another important finding of our study was that children who suffered from illnesses were more vulnerable to CFM. Diseases related to undernutrition can progress to acute or chronic conditions. This mostly occurs due to energy imbalance, high catabolism rate, decreased appetite and increased energy consumption^(34,44)^. So, repeated illness can progress to undernutrition in children. A similar finding was reported by Sumon *et al.*, who found a significant association between CFM and childhood illness^(38)^.

As a nationally representative sampling method was used in both surveys, this study can be considered to have painted the actual picture of the burden of CFM. Although a few studies have been conducted before focusing on the existence of multiple nutritional disorders, the definition of different types of malnutrition in two different time periods is unique to this study. However, it also had some limitations. Due to the cross-sectional nature of the study design, establishing a causal relationship was not possible. Key variables, like food history, maternal malnutrition, trend etc., were not considered due to unavailability, which would have been interesting to explore.

### Conclusion

The current study indicates that malnutrition is still a colossal public health concern in an LMIC like Bangladesh. The study findings evaluated the prevalence, trends and several determinants of CFM among children in the country. This article should capture the attention of researchers, policymakers and stakeholders regarding the CFM, a topic that is considered minor in the field of nutrition research. The authors propose further prospective studies to investigate the outcomes of CFM in community as well as hospital settings. In light of the findings, the authors recommend enhancing maternal education programmes and providing financial assistance to vulnerable families that can improve child nutrition. Scaling up initiatives like the National Nutrition Service and public awareness campaigns by leveraging media to promote knowledge of nutrition, hygiene and healthcare-seeking behaviours can reduce the burden of SFM and CFM. Collaboration between multiple sectors can prove to be a beneficial way forward in achieving sustainable reductions in childhood malnutrition.
